# Neonatal herpes simplex virus encephalitis: a single-center retrospective study of 14 cases

**DOI:** 10.3389/fped.2026.1740937

**Published:** 2026-03-31

**Authors:** Lu Liu, Le Wang, Peng Zhang, Mingyu Gan, Ruixuan Liujiang, Guoqiang Cheng, Mengmeng Ge

**Affiliations:** 1 Pediatrics Department of Shanghai Fifth People’s Hospital, Fudan University Affiliated, Shanghai, China; 2 Neonatal Intensive Care Unit, NICU, Xi'an Children’s Hospital, Xi'an, China; 3 Key Laboratory of Neonatal Disease, Ministry of Health, Children’s Hospital of Fudan University, Shanghai, China; 4 Clinical Genetic Center, Children’s Hospital of Fudan University, Shanghai, China; 5 Department of Neonatology, International Peace Maternity and Child Health Hospital, Shanghai Jiao Tong University School of Medicine, Shanghai, China; 6 Department of Neonates, Children’s Hospital, Fudan University, Shanghai, China

**Keywords:** acyclovir, diagnosis, encephalitis, herpes simplex, infant, newborn, manifestations, prognosis

## Abstract

**Background:**

This single-center retrospective study aims to analyze the clinical characteristics, treatment strategies, and outcome at discharge of neonatal-onset herpes simplex virus encephalitis (NHSE).

**Methods:**

We conducted a single-center retrospective case review of infants diagnosed with NHSE at the Children's Hospital of Fudan University between February 1, 2016, and February 1, 2024. Clinical data, including demographics, clinical symptoms, laboratory findings, neuroimaging results, treatment regimens, and outcomes at discharge, were collected and analyzed.

**Results:**

A total of 14 infants with NHSE (7 males, 7 females) were identified at our center, with a median age at diagnosis of 26 days (range: 7–51 days). Initial symptoms predominantly included fever and seizures, with neurological involvement (e.g., seizures, lethargy, irritability or altered mental states) in 13 cases. Physical examinations, such as bulging anterior fontanel, were noted. Herpes simplex virus (HSV)-DNA was detected in 13 cases (6 HSV-1, 7 HSV-2) through cerebrospinal fluid (CSF) polymerase chain reaction (PCR) or metagenomic testing. Among these, 9 cases were identified via CSF-PCR, with 7 testing positive on the initial examination and 2 on repeated testing. Notably, 6 cases were diagnosed using metagenomic next-generation sequencing (mNGS), all of which yielded positive results on the first test. Ten out of the 12 children often exhibited temporal lobe spikes on video electroencephalograms (VEEGs). Early magnetic resonance imaging (MRI) revealed cytotoxic edema, progressing to multicystic encephalomalacia. All received acyclovir antiviral treatment. Seven discontinued treatments, one was referred for ocular lesions, and six improved and were discharged.

**Conclusions:**

In this single-center cohort, NHSE often presents with nonspecific fever and seizures, with late onset and absent indicative rashes, complicating early diagnosis. For newborns suspected of having NHSE, early CSF HSV-DNA testing and prompt antiviral treatment are essential to improve outcomes. Metagenomic sequencing is especially valuable for accurate, rapid diagnosis when conventional methods fail.

## 1 Introduction

Neonatal-onset herpes simplex virus encephalitis (NHSE) is a severe infectious disease that significantly affects the prognosis of the central nervous system, often resulting in serious complications or death, with many survivors suffering from long-term sequelae (1). Advances in diagnostic technologies and the widespread use of acyclovir have significantly improved the prognosis of NHSE (1). However, the nonspecific clinical manifestations and relatively late onset during the neonatal period frequently lead to misdiagnoses and missed diagnoses. Delayed diagnosis often results in the failure to initiate timely and effective treatment, thereby adversely affecting patient outcomes (2). In this single-center retrospective study, we reviewed the medical records of infants diagnosed with NHSE at the Children's Hospital of Fudan University. We analyzed their clinical characteristics, laboratory findings, neuroimaging results, treatment processes, and outcomes at discharge. The objective was to deepen understanding of NHSE, promote early diagnosis, and facilitate timely treatment to improve outcomes in affected neonates.

## 2 Methods

### 2.1 Inclusion criteria

1. This single-center retrospective study was conducted on infants with NHSE admitted to the Department of Neonatology, Children's Hospital affiliated with Fudan University, between February 1, 2016, and February 1, 2024. Inclusion criteria were as follows:
2. Age at symptom onset ≤28 days;
3. Evidences of confirmed or highly suspected NHSE (meeting at least one of the following sub - criteria):
  1. Laboratory-confirmed Herpes simplex virus (HSV) infection in cerebrospinal fluid (CSF) during hospitalization, evidenced by any of the following: HSV-DNA positivity, conversion of HSV-IgM from negative to positive, initial HSV antibody positivity, or detection of HSV by metagenomic next-generation sequencing (mNGS);
  2. Clinical manifestations indicative of neonatal HSV infection, including seizures, abnormal neurological findings, abnormal video electroencephalograms (VEEGs), and/or neuroimaging abnormalities, accompanied by one or both of the following: isolation of HSV from surface cultures or swabs collected from skin lesions performed 12–24 h after birth, or detection of HSV in plasma by polymerase chain reaction (PCR) or mNGS.

Patients with genetic metabolic disorders, neonatal hypoxic-ischemic encephalopathy, intracranial masses, cranial trauma, cerebral infarction, or other bacterial or viral infectious neurological disorders were excluded.

### 2.2 Collection of clinical data

1. General information: sex, gestational age, birth weight, mode of delivery, Apgar score, and history of maternal infection during pregnancy.
2. Clinical features: age at onset, age at diagnosis, neurological manifestations, involvement of skin, eyes, and mouth (SEM).
3. Laboratory tests:
  1. Blood tests: White blood cell count (WBC), lymphocyte percentage (L%), C-reactive protein (CRP) and platelet count (PLT), measured on admission; HSV-DNA and HSV-specific IgM antibodies during hospitalization.
  2. CSF analysis: WBC, (L%), red blood cell count (RBC), glucose (Glu), chloride (Cl⁻), protein levels, HSV-DNA and HSV-specific IgM antibodies during hospitalization.
4. Laboratory Diagnostics:
  1. Serological Testing: Serum and CSF samples were tested for HSV-specific IgM antibodies using a commercially available chemiluminescence immunoassay (CLIA) kit (LIAISON® HSV-1/2 IgM, DiaSorin Italia S.p.A., Saluggia, Italy). The assay was performed and interpreted in strict accordance with the manufacturer's instructions. It is important to note that this IgM assay is a pan-HSV test and cannot differentiate between HSV-1 and HSV-2 specific antibodies.
  2. Molecular Detection and Typing: Viral DNA was extracted from CSF and lesion swab samples using the Nucleic Acid Extraction or Purification Kit (DAAN GENE Co., Ltd., Guangzhou, China). Detection and differentiation of HSV-1 and HSV-2 DNA were performed using commercial multiplex real-time PCR assays. Specifically, the Herpes Simplex Virus Type 1 Nucleic Acid Detection Kit (Baitai Gene Engineering Co., Ltd., Wuhan, China) and the Herpes Simplex Virus Type 2 Nucleic Acid Determination Kit (DAAN GENE Co., Ltd., Guangzhou, China) were used according to the manufacturers’ protocols. Amplification and detection were conducted on an ABI 7500 Real-Time PCR System (Applied Biosystems, Foster City, CA, USA). A sample was considered positive if the cycle threshold (Ct) value was ≤37.
5. Auxiliary examinations: brain computed tomography (CT), brain MRI, VEEGs, visual evoked potential testing, and auditory evoked potential testing.

Neuroimaging was performed as clinically indicated. MRI, including diffusion-weighted sequences, served as the primary and preferred modality at our center for characterizing cerebral injury. In a subset of neonates-mostly examined at referring hospitals prior to transfer-CT was employed in acute situations where MRI was not immediately available or when rapid structural information was urgently needed to guide clinical decisions (e.g., to exclude neurosurgical emergencies).

6. Antiviral treatment.
7. Outcome at discharge.

### 2.3 Statistical analysis method

Statistical analysis was conducted using SPSS version 22.0 software. Measurement data conforming to a normal distribution were expressed as mean ± standard deviation (*x* ± *s*), while data with a skewed distribution were represented as the median (range). Categorical data were described as frequencies (proportions).

## 3 Results

### 3.1 Basic information

During the study period, 14 cases of NHSE were identified, consisting of 7 males and 7 females (see Table 1 for details). Of these, 13 cases were full-term infants, while one case was a preterm infant. The mean birth weight was 3,252 ± 422 g, with one case (Case 8) classified as small for gestational age. Twelve cases were delivered vaginally, while 2 cases required conversion to cesarean section due to “failed trial of labor” (Cases 3 and 9). Four cases presented with normal Apgar scores at birth, while one case (Case 3) had a history of severe asphyxia. Maternal infection history during pregnancy was reported in 3 cases, with only one case (Case 6) suspected of herpes infection. No evidence of HSV infection was identified during pregnancy in the remaining 13 cases.

**Table 1:** Demographic and clinical characteristics of 14 infants with NHSE at a single tertiary center in China (2016–2024).

Case	Gender	Gestationalage (w)	Birthweight (g)	Deliverymode	Apgarscore	Prenatalinfection	Onsetage (day)	Diagnosisage (day)	Initialsymptoms	Neurologicalmanifestations	SEM		
											Skin	Eye	Oral
1	Male	40	3,200	Vaginal	10-10	Urethritis, Vaginitis	14	25	Fever (38.6 °C)	Bulging fontanelle, separated sutures	None	Retinal vasculitis	None
2	Male	39^+5^	3,450	Vaginal	10-10	None	5	20	Poor feeding, lethargy	Lethargy, seizures	None	Retinal vasculitis	None
3	Male	40^+1^	3,415	Cesarean	3–7	None	1	7	Seizures	Seizures, irritability	None	Retinal vasculitis	None
4	Female	39^+1^	3,750	Vaginal	Unknown	None	23	34	Fever (maximum temperature unknown)seizures	Seizures	None	Chorioretinitis	None
5	Male	40^+3^	3,350	Vaginal	Unknown	None	16	35	Poor feeding, seizures	Seizures	None	Fundushemorrhage	None
6	Female	41^+1^	3,320	Vaginal	Unknown	Suspected herpes infection	14	27	Fever (maximum temperature unknown)	Bulging fontanelle	Red rash	Chorioretinitis	None
7	Female	38	2,900	Vaginal	Unknown	None	11	22	Fever (38.4 °C) poor feeding	Seizures, lethargy	Rash desquamation	Not tested	None
8	Male	39^+5^	2,800	Vaginal	Unknown	Mycoplasma infection	17	43	Fever (38.7 °C) seizures	Seizures, lethargy, irritability, bulging fontanelle	None	Chorioretinitis	None
9	Male	38^+3^	3,300	Cesarean	10-10	None	22	51	Fever (maximum temperature unknown) poor mental state	Poor mental state	None	None	None
10	Female	40^+3^	3,550	Vaginal	Unknown	None	25	28	Fever (37.8 °C) seizures	Seizures	None	None	None
11	Male	38^+3^	3,350	Vaginal	Unknown	None	15	23	Fever (38.6 °C)	Poor mental state	None	None	None
12	Female	33^+6^	2,070	Vaginal	Unknown	None	1	18	Poor feeding, vomiting	None	None	None	None
13	Female	39^+1^	3,500	Vaginal	Unknown	None	26	35	Fever (39.3 °C)	Seizures	None	None	None
14	Female	41^+1^	3,580	Vaginal	10-10	None	11	17	Seizures	Seizures	None	None	None

### 3.2 Clinical features

The detailed clinical features of the 14 cases are shown in Table 1. The median age of onset was 15 days (range: 1–26 days), and the median age at diagnosis was 26 days (range: 7–51 days). The primary initial symptoms were fever (9 cases) and seizures (6 cases), with peak temperatures predominantly classified as moderate fever. During the disease course, 10 cases exhibited fever, and 13 cases demonstrated clinical features of neurological involvement, including seizures (9 cases), lethargy (3 cases), irritability (2 cases), and impaired mental state (2 cases). Abnormal physical examination findings included three cases with bulging fontanelle or fontanelle protuberance and one case with cranial suture separation. Notably, two infants (Cases 1 and 6) exhibited abnormal neurological signs on physical examination in the absence of classic clinical symptoms such as seizures or altered consciousness, None of the cases exhibited signs of oral mucosal involvement. Two cases developed rashes, although these did not show typical herpes-like characteristics. Ocular manifestations were observed in seven cases, including three cases of retinal vasculitis, three cases of choroidoretinitis, and one case of fundus hemorrhage. Six cases exhibited normal ocular findings, while one infant (Case 7) did not undergo an ophthalmological examination due to severe illness and treatment discontinuation.

### 3.3 Laboratory tests

Laboratory test results for 14 patients are summarized in Table 2. Thirteen patients underwent routine blood tests upon admission, with a median WBC count of 6.6 × 10^9^/L (range: 3.82–19.8 × 10^9^/L). Among these, four patients had elevated WBC counts, whereas one patient exhibited a decreased WBC count. Lymphocyte percentage ranged from 32.2% to 79.2%. The median platelet count was 283 × 10^9^/L (range: 120–449 × 10^9^/L), with five patients exhibiting elevated platelet counts. Four patients exhibited elevated CRP levels. Initial CSF analysis upon admission showed normal WBC counts in eight of the 14 patients, while six patients showed elevated WBC counts, with a median WBC count of 14 × 10^6^/L (range: 0–170 × 10^6^/L). Lymphocytes predominated in the white blood cell population, with lymphocyte percentages ranging from 60% to 90%. Two out of the 14 patients had elevated elevated RBC counts, with a median RBC count was 2.8 × 10^6^/L (range: 0–7 × 10^6^/L). Seven out of the 14 patients had elevated protein levels, with a median protein content of 1,899 mg/L (range: 493.6–5,464.7 mg/L). Eight out of the 14 patients had decreased glucose levels, while one exhibited an elevated glucose level. The median glucose content was 1.9 mmol/L (range: 1.0–5.2 mmol/L). Thirteen out of the 14 patients tested positive for HSV-DNA in the CSF, including seven with HSV-1 DNA and six with HSV-2 DNA. Among these, nine patients were diagnosed through CSF HSV-PCR testing, with seven diagnosed on the first test and two on repeated testing. Additionally, six patients with positive CSF HSV-DNA were diagnosed through CSF mNGS, all showing positive results on the first test. Only two patients of these patients underwent concurrent blood mNGS, which also returned positive results. Notably, one patient was diagnosed based on the conversion of CSF HSV-IgM antibodies from negative (5 days post-onset) to positive (10 days post-onset), although the HSV type was specified. In serum testing, four out of the 9 patients tested positive for HSV-IgM, and one out of the 7 patients tested positive for HSV-DNA.

**Table 2:** Laboratory and virological findings in 14 infants with NHSE at a single tertiary center in China (2016–2024).

Case	Blood routine				CSF								Serum		Metagenomics
	WBC (×10^9^/L)	L%	CPR (mg/L)	PLT (×10^9^/L)	WBC (×10^6^/L)	L%	RBC (×10^6^/L)	Cl (mmol/L)	Glu (mmol/L)	Protein (mg/L)	HSV-IgM	HSV-DNA	HSV-IgM	HSV-DNA	HSV-DNA
1	12.7	46.3	<8	421	120	68	2	124	1.3	2,164	Negative	Negative	HSV1/2-IgM+	Negative	Not tested
	10.8	42.6		394	170	82	3	123	1.2	4,677	HSV1/2-IgM+	Negative			
2	5.8	46.1	<8	311	0	Unknown	Unknown	102	1	1,737	Not tested	Negative	Negative	Negative	Not tested
	6.6	67.8		396	105	60	1	126	1.9	3,122	Not tested	HSV2-DNA+			
3	19.8	45.8	<8	283	8	Unknown	0	118	2.5	500	Not tested	HSV1-DNA+	Negative	Not tested	Not tested
4	7.1	44.3	<8	263	8	Unknown	Unknown	118	2.5	500	Not tested	HSV2-DNA+	Negative	HSV2-DNA+	Not tested
5	6	44.7	12	363	50	80	4	120	1.5	2,062	Not tested	Negative	HSV1/2-IgM+	Not tested	Not tested
	6.2	74.1	<8	408	20	90	5	124	1.7	3,671	Not tested	HSV2-DNA+			
6	6	67.3	<8	417	124	80	7	126	1.2	3,372	Not tested	HSV2-DNA+	HSV1/2-IgM+	Negative	Not tested
7	11.1	32.2	28	245	50	84	2	125	2.1	5,464.7	Not tested	HSV2-DNA+	Negative	Negative	CSF-HSV2-DNA+
8	5.9	63.9	<8	449	0	Unknown	Unknown	129	1.3	3,223	Not tested	HSV1-DNA+	HSV1/2-IgM+	Negative	Blood & CSF-HSV-DNA+
9	4.5	38.9	14	213	5	70	1	124	2.2	621.5	Not tested	HSV2-DNA+	Negative	Negative	Not tested
10	5.5	63.5	<8	265	8	Unknown	0	129	1.6	3,245.5	Not tested	Negative	Not tested	Not tested	Blood & CSF-HSV-DNA+
11	3.82	60.1	54.2	120	42	70	3	120.3	3.59	850	Not tested	Negative	Not tested	Not tested	CSF-HSV1-DNA+
12	11.5	58.3	<8	157	0	Unknown	0	120	3.1	493.6	Not tested	Negative	Not Tested	Not tested	CSF-HSV1-DNA+
13	7.76	79.2	<8	185	0	Unknown	Unknown	114	5.2	707.6	Not tested	Negative	Not Tested	Not tested	CSF-HSV1-DNA+
14	8.5	65	<8	173	9	Unknown	0	116	2.7	732.1	Negative	HSV1-DNA+	Negative	Negative	Not tested

WBC, the normal reference range in blood routine tests is 4–10 × 10^9^/L, while in cerebrospinal fluid it is 0–15 × 10^6^/L. CRP, the normal reference range is <8 mg/L. PLT, the normal reference range is 100–300 × 10^9^/L. Cerebrospinal fluid biochemistry: the normal reference range for RBC in is <5 × 10^6^/L, chloride is 120–132 mmol/L, for glucose is 2.5–4.4 mmol/L, and for protein is 400–1,200 mg/L.

### 3.4 Auxiliary examinations

**Figure 1:**
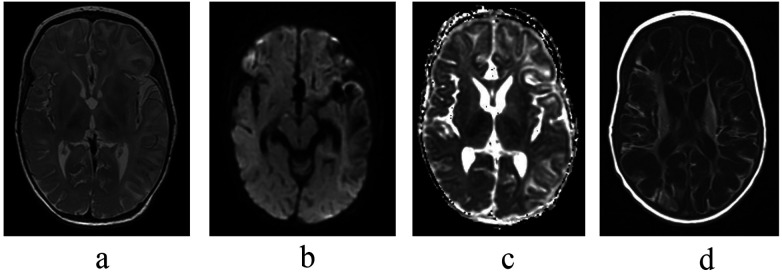
Serial brain MRI findings in an infant with NHSE (case 14), showing progression from early cytotoxic edema to multicystic encephalomalacia **(a–c)** acute-stage findings (Day 5 after symptom onset). Axial images demonstrate the severe, diffuse nature of early NHSE, featuring widespread cytotoxic edema involving the cerebral hemispheres and thalami. **(a)** T2-weighted image shows diffuse cortical swelling and hyperintensity with profound blurring of the gray-white matter differentiation, indicating extensive vasogenic edema. **(b)** DWI reveals confluent areas of marked hyperintensity throughout the affected deep and cortical structures, confirming widespread restricted diffusion due to acute cytotoxic edema. **(c)** Apparent Diffusion Coefficient (ADC) map provides definitive pathophysiological confirmation, showing corresponding pronounced hypotensity that delineates the full extent of cellular injury. **(d)** End-stage sequelae (Day 20 after symptom onset; follow-up 15 days after initial MRI). Follow-up axial T1-weighted image reveals the devastating progression to extensive parenchymal destruction. The cerebral architecture is largely replaced by confluent areas of hemorrhagic parenchymal softening and early cystic encephalomalacia, accompanied by significant cerebral volume loss (atrophy), representing the characteristic final stage of severe NHSE.

The auxiliary examination results for 14 children are summarized in Table 3. Twelve children underwent VEEG testing of which two were normal, while nine showed epileptiform discharges (Cases 1, 2, 5, 6, 7, 8, 10, 11, 14). Persistent low voltage was observed in four cases (Cases 4, 5, 7, 8). Brain imaging findings were as follows. Eight out of the 14 children underwent brain CT scans during the early stage of the disease (within 1 week), all of which indicated cytotoxic edema. Of these eight, one case primarily involved the frontal and parietal lobes (Case 4), while two cases primarily affected the frontal and temporal lobes (Cases 5, 7). Thirteen children underwent brain MRI, with one showing normal findings and twelve demonstrating cytotoxic edema, hemorrhagic lesions, or destructive lesions during the early stage of the disease (median age: 7 days). Bilateral multifocal or diffuse involvement was observed, particularly in the frontal, parietal, and temporal lobes, as well as the thalamus. The corpus callosum and posterior limbs of the internal capsule were also frequently affected. In the later stage (median age: 19 days), diffuse multicystic encephalomalacia developed in five infants (as shown in Figure 1). Nine children underwent auditory evoked potential testing, with seven demonstrating normal hearing and two showing mild hearing loss. Only three children underwent visual evoked potential testing, and all showed abnormalities.

**Table 3:** Neuroimaging and electrophysiological findings in 14 infants with NHSE at a single tertiary center in China (2016–2024).

Case	VEEG findings	HCT findings	MRI findings	Auditory evoked potential (AEP)	Visual evoked potential (VEP) (VEP)
1	16 days: Seizure-like discharges, predominantly in the anterior region	Not tested	11 days: Hemorrhagic lesion in the left temporal lobe, abnormal signals in bilateral parietal and right temporal lobes, corpus callosum	Normal	No significant waveform elicited
			32 days: Bilateral temporal and parietal encephalomalacia with gliosis, abnormal signals in dorsal thalami		
2	34 days: Seizure-like discharges, paroxysmal slow electrical activity	8 days: Diffuse density reduction	10 days: Multifocal traumatic lesions (bilateral frontal, temporal, parietal, occipital lobes, basal ganglia, thalamus, internal capsule, corpus callosum)	Normal	Not tested
3	Normal	Not tested	7 days: Multifocal abnormal signals (bilateral thalami, periventricular regions, semioval centers, right frontal-parietal cortical T1W1 hyperintensity)	Normal	Not tested
			27 days: Abnormal signals reduced; suspected T1W1 signals in bilateral thalami; myelination signals in posterior limbs of internal capsule		
4	12 days: Persistent low voltage	2 days: Bilateral frontal and parietal hypodensity, suspected cytotoxic edema	5 days: Multifocal hemispheric lesions, predominantly in the left hemisphere	Mild hearing loss in the left ear	Not tested
			13 days: Multidirectional low signals in the right cerebellar hemisphere		
5	1–6 days: Low voltage in the central-parietal/temporal-parietal anterior region; continuous seizures	1–6 days: Hypodensity in the right frontal-temporal lobe	7 days: Diffuse cortical swelling, multiple DWI hyperintensities in basal ganglia, thalamus, corpus callosum, blurred gray-white differentiation	Mild bilateral hearing loss	Not tested
			14 days: Extensive cerebral damage including the brainstem, cystic encephalomalacia in the thalamus, and gliosis		
6	14 days: Sharp waves and slow waves in bilateral frontal-parietal regions during sleep	Not tested	11 days: Reduced white matter fibers in the left temporal lobe	Normal	Bilateral amplitude reduction, poorly differentiated waveforms, prolonged latency
			17 days: Multifocal encephalomalacia with atrophy in the left frontal-temporal region		
7	4 days: Persistent low voltage, low-moderate amplitude slow waves and sharp waves	2 days: Blurred gray-white differentiation, diffuse hypodensity in bilateral hemispheres, prominent in frontal and temporal lobes, patchy low-density thalamic signals	6 days: Diffuse traumatic lesions; multifocal T1W1 hyperintensities in the periventricular, occipital, and right temporo-occipital junction	Not tested	Not tested
			19 days: Worsened diffuse parenchymal changes		
8	1 days: Persistent low voltage, sharp and slow waves predominantly in left frontal and middle temporal regions	1 days: cytotoxic edema	11 days: Diffuse hemispheric and thalamic lesions; supratentorial ventriculomegaly	Normal	Right eye: Low amplitude, prolonged latency, Poor differentiatio Left eye: no response
			19 days: Extensive supratentorial tissue loss; multicystic encephalomalacia		
9	Normal	Not tested	6 days: Subdural hematoma in the posterior fossa (prominent on the left side)	Normal	Not tested
10	4 days: Severe abnormalities with frequent discharges, burst-suppression pattern	1 days: cytotoxic edema	2 days: Multifocal cortical abnormalities in bilateral frontal-temporal-parietal lobes and thalami; diffusion restriction	Not tested	Not tested
			11 days: Widespread abnormal signals, forming softening foci		
			28 days: Persistent diffuse abnormal signals and multicystic encephalomalacia		
11	15 days: Moderate abnormalities, with frequent multifocal sharp and slow waves during sleep	2 days: Blurred gray-white differentiation, diffuse hypodensity in white matter, widened grooves in frontal, parietal, temporal lobes	5 days: Low T1WI signals, high T2WI signals in bilateral hemispheric white matter	Not tested	Not tested
12	Not tested	Not tested	28 days: Premature infant brain changes: MRI appears appropriate for corrected gestational age, showing expected immature gyral pattern and myelination without focal lesions	Not tested	Not tested
13	Not tested	Not tested	Not tested	Not tested	Not tested
14	6 days: Severe neonatal EEG abnormalities with burst-suppression, frequent multifocal seizures	1 days: Diffuse white matter hypodensity	5 days: Diffuse hemispheric and thalamic abnormalities; acute injury signs with mild subdural hematoma; sagittal sinus narrowing	Normal	Not tested
			20 days: Extensive parenchymal injury, hemorrhagic softening, partial atrophy		

### 3.5 Treatment and outcome at discharge

All 14 children in this study received antiviral treatment. Seven out of the 14 children received acyclovir immediately after diagnosis at a dose of 20 mg/kg per administration, administered intravenously three times daily, for a median duration of 21 days (range: 14–30 days). Six children initiated acyclovir treatment prior to diagnosis based on clinical symptoms and auxiliary examination findings suggestive of possible viral encephalitis. During the treatment process, seven children were discharged against medical advice (DAMA), among whom three developed encephalitis sequelae, presenting as difficulty in sucking, delayed responses, and recurrent seizures. One child (Case 6) required surgical intervention for significant ocular lesions and was referred to another hospital. The remaining six children were discharged after completing antiviral treatment, with CSF retesting confirming negative HSV-DNA results.

## 4 Discussion

This single-center retrospective study, conducted at a national children's medical center, elucidates the clinical profile, diagnostic hurdles, and management realities of neonatal herpes simplex encephalitis (NHSE). It highlights the systemic challenges and proposes actionable strategies for optimizing care within a tiered healthcare framework.

HSV is a critical etiological agent of severe encephalitis in neonates (3, 20). In our cohort of 14 infants with NHSE, the median age at symptom onset was 15 days, consistent with the established epidemiological window. However, the median age at confirmed diagnosis was 26 days, revealing a substantial diagnostic delay of 11 days. This delay is primarily attributable to the non-specific initial presentation: the majority of infants presented with common symptoms such as fever and seizures, and characteristic herpetic skin lesions were absent in 12 cases (85.7%). These features frequently lead to initial misdiagnosis as neonatal sepsis or other forms of meningitis, underscoring the necessity of including NHSE in the primary differential diagnosis for any neonate with unexplained neurological signs (4, 5). Compounding this diagnostic challenge, a documented maternal history of HSV infection was lacking in 13 cases, aligning with the evidence that a significant proportion (up to two-thirds) of prenatal HSV infections are asymptomatic or non-specific (6, 7). Therefore, in clinical practice, the absence of identifiable perinatal risk factors should not diminish vigilance for NHSE.

CSF HSV-DNA PCR remains the gold standard for confirming NHSE and determining viral type (8, 9). However, its sensitivity is limited in early infection, as reflected in our cohort where only slightly over half of confirmed cases were diagnosed on the first lumbar puncture-a delay often due to low viral load or prior antiviral treatment (10). This diagnostic gap underscores the utility of mNGS. In our neonatal cohort, CSF mNGS achieved 100% detection in applied cases, identifying HSV even in some initially PCR-negative patients. As a non-hypothesis-driven, broad-spectrum detection tool, mNGS shows unique advantages in diagnosing encephalitis of unknown etiology, in critically ill patients, or when conventional tests are negative (11). Nevertheless, current constraints including cost and analytical complexity position it as a complementary adjunct to PCR, not a replacement (12). We propose an integrated pathway: initiate empirical therapy and urgent CSF HSV-PCR for suspected cases; reserve mNGS for those with high clinical suspicion but negative or inconclusive PCR, or when rapid comprehensive pathogen screening is required. Prospective studies are needed to define the impact of mNGS on clinical outcomes and cost-effectiveness.

Our findings underscore distinct neurodiagnostic profiles between neonatal and childhood-onset HSE. demonstrates high sensitivity for NHSE, often revealing abnormalities before structural changes are apparent on CT or MRI, especially early in infection (13). In neonates, these abnormalities are primarily characterized by focal or multifocal periodic/episodic discharges (e.g., periodic discharges, burst-suppression or electrographic seizures), contrasting with the classic temporal lobe spikes and slow waves typical in older children. This difference likely stems from the more diffuse and multifocal nature of neonatal brain injury. Consequently, any focal or multifocal epileptiform activity in a neonate with acute encephalopathy should prompt urgent suspicion for NHSE and virological investigation (14).

Neuroimaging in NHSE follows a dynamic temporal profile, evolving from initially normal studies to characteristic patterns of injury. Within the first week post-onset, MRI often reveals cytotoxic edema, hemorrhagic foci, and early destructive changes, which may be focal (including temporal) or diffuse (15). In severe or advanced cases, typically beyond 2 weeks, imaging progresses to parenchymal softening, cystic degeneration, and ultimately cerebral atrophy or multicystic encephalomalacia (16). Our data corroborate this timeline: among 13 infants with serial MRI, the median time to detectable cytotoxic edema was 7 days (range: 2–11), with cerebral softening observed at a median of 19 days (range: 11–32). Notably, diffusion-weighted imaging (DWI) proved most sensitive for early detection, identifying edema as early as 5 days post-onset in two cases, underscoring its critical role in the initial evaluation of suspected NHSE (17).

Acyclovir is the first-line treatment for NHSE. Clinical guidelines strongly advocate for the immediate initiation of empirical therapy upon suspicion of NHSE, prior to virological confirmation, to mitigate the high risk of poor outcomes associated with treatment delay (18). While this approach is widely advised for neonates with poor general health, fever or aseptic meningitis, the decision is less straightforward in otherwise well-appearing neonates-such as those with isolated CSF mononuclear pleocytosis or fever without localizing signs-where risks, benefits and cost-effectiveness must be carefully weighed (18, 19). Our findings from a national referral center reflect this clinical complexity. All patients received intravenous acyclovir, yet the timing of initiation varied: empirical therapy was promptly started based on high clinical suspicion in a subset of cases, while for others, documented treatment began at or after confirmatory testing. This variation underscores the essential role of bedside judgment in applying guidelines. Furthermore, the recorded interval between symptom onset and formal diagnosis must be interpreted within the context of a tiered healthcare system. For inter-regionally referred infants, this interval incorporates unavoidable delays related to transfer logistics and the subsequent completion of definitive testing at the tertiary facility. Thus, the documented time of diagnosis often lags substantially behind the point of clinical suspicion and empirical treatment initiation. No clinically significant difference in short-term discharge outcomes was observed between these timing approaches in our cohort, though the small sample size and inherent disease severity preclude definitive conclusions-highlighting the need for larger prospective studies.

The long-term neurological burden of severe NHSE remains inadequately characterized, in part due to fragmented post-discharge follow-up (6). Our study reveals a critical, underreported challenge in the management of NHSE within a tiered referral system: a substantial rate of DAMA, with most infants in this subgroup already exhibiting severe and likely irreversible neurological sequelae at discharge. This observation highlights that poor outcomes are driven not only by the intrinsic neurovirulence of HSV but also by socioeconomic barriers and disruptions in the continuum of care. A key limitation of this retrospective analysis is the consequent absence of systematic long-term neurodevelopmental data, a direct reflection of the high rate of loss to follow-up typical of national referral centers. This evidence gap fundamentally constrains a complete understanding of the disease's natural history and underscores the urgent need for prospective, multicenter cohorts with standardized, protocol-driven long-term follow-up. Only through such coordinated efforts can accurate outcome trajectories be delineated and modifiable prognostic factors-both biological and systemic-be identified to guide comprehensive management strategies.

## 5 Conclusions

In summary, this single-center retrospective study demonstrates that NHSE is a severe condition that can result in significant neurological complications. Clinically, NHSE is characterized by initial symptoms such as fever and seizures, the appearance of herpetic rash during the disease course, CSF findings indicative of aseptic meningitis with increased lymphocyte cells, a VEEG showing focal or multifocal periodic/episodic discharges in the early stages, or brain MRI with DWI sequences revealing cytotoxic edema within the first week of disease onset, while signs of encephalomalacia emerging after 2–3 weeks of follow-up, strongly suggesting NHSE. Moreover, CSF mNGS plays a critical role in the early diagnosis of NHSE.

## Data Availability

The original contributions presented in the study are included in the article/Supplementary Material, further inquiries can be directed to the corresponding author.
